# Clinical and Radiological Outcomes of Gustilo-Anderson Type IIIB Open Fractures in 125 Patients

**DOI:** 10.7759/cureus.35441

**Published:** 2023-02-25

**Authors:** Almigdad Ali, Ahmed Aljawadi, Ibrahim H Elkhidir, Camille De-Shoulepnikoff, Anand Pillai

**Affiliations:** 1 Trauma and Orthopaedics, NHS (National Health Service) Greater Glasgow and Clyde, Glasgow, GBR; 2 Radiology, Betsi Cadwaladr University Health Board, Bangor, GBR; 3 Faculty of Medicine, University of Khartoum, Khartoum, SDN; 4 Orthopaedics, Centre Hospitalier Universitaire Vaudois, Lausanne, CHE; 5 Trauma and Orthopaedics, Wythenshawe Hospital, Manchester, GBR

**Keywords:** gustilo-anderson, infection, union, debridement, orthoplastic, open fracture

## Abstract

Introduction: This study describes single-centre outcomes of Gustilo-Anderson type IIIB open fractures in relation to the current standards in the United Kingdom, which aim at performing skeletal fixation and soft tissue coverage at an early stage to salvage the limb and achieve bone union with a minimum infection rate. Methods: A total of 125 patients with 134 Gustilo-Anderson type IIIB open fractures, who had definitive skeletal fixation with soft tissue coverage between June 2013 and October 2021, were prospectively followed up and included in this study.

Results: Initial debridement was performed within 12 hours from the time of injury for 62 (49.6%) patients and within 24 hours for 119 (95.2%) patients (mean= 12.4 hours). Definitive skeletal fixation and soft tissue coverage were achieved within 72 hours for 25 (20%) patients and within seven days for 71 (57%) patients (mean= 8.5 days). The mean follow-up duration was 43.3 (6-100) months, and the limb salvage rate was 97.1%. The occurrence of deep infections was associated with time from injury to initial debridement (p=0.049). Three patients (2.4%) developed deep (metalwork) infections, all three had their initial debridement performed within 12 hours from the time of injury. There was no association between time to definitive surgery and the development of deep infection (p=0.340). Bone union was achieved in 84.3% of patients following their primary surgery. Time to union was associated with fixation modality (p=0.002) and type of soft tissue coverage (p=0.028), and was negatively correlated with time to initial debridement (p=0.002, correlation coefficient -0.321). There was a 0.27-month decrease in time to union for every hour delay in time to debridement (p=0.021).

Conclusion: Delaying initial debridement or definitive fixation and soft tissue coverage didn’t increase the rate of deep (metalwork) infections. The time to achieve bone union was negatively correlated with the time from injury to initial debridement. We advise prioritising surgical technique and availability of expertise over strict adherence to time thresholds of surgeries.

## Introduction

Open fractures are complex injuries, usually resulting from high-energy trauma [1]. They are a cause of significant morbidity and mortality, and require a multidisciplinary approach in their management [2]. Gustilo-Anderson (GA) classified open fractures into three main types based on wound size, severity of soft tissue/bony injuries, and level of contamination [3]. GA type IIIB open fractures are associated with significant soft tissue loss and require soft tissue coverage in addition to skeletal stabilisation [4]. Complications of these fractures are mainly linked to deep infections and non-union, both of which can result in the limb being unsalvageable and are costly for both the patient and the health system [5].

The British Orthopaedic Association (BOA) and the British Association of Plastics, Reconstructive and Aesthetic Surgeons (BAPRAS) published standards for practice in 2017 outlining the management of patients with open fractures. These standards aimed at enabling optimum recovery and minimising the risk of infection through early antibiotic administration, timely thorough surgical debridement, and simultaneous skeletal fixation and soft-tissue coverage [6]. This orthoplastic approach of simultaneous skeletal fixation and soft-tissue cover has reportedly yielded better outcomes in regards to infection, time to union, and soft tissue recovery in settings both inside and outside the UK (both developed and developing countries) [2,7,8].

This study evaluates the outcomes of a combined orthoplastic approach in managing GA type IIIB open fractures in an orthoplastic unit in Manchester, building on a previously published paper in our institution, with a larger sample size and an extended follow-up duration [9].

## Materials and methods

The study was conducted at Wythenshawe Hospital, Manchester, England. All patients who were admitted to our orthoplastic unit with GA type IIIB open fractures from June 2013 to October 2021, and had a single-stage “fix-and-flap” orthoplastic surgery, were prospectively followed up and included in this study. These patients were either admitted from our emergency department (ED) or referred from regional hospitals after initial debridement and temporary stabilisation, with or without the application of negative pressure dressing.

After initial management in ED according to BOA/BAPRAS guidelines for the management of open fractures [6], patients were either booked into the emergency theatre or placed on the soonest trauma list for initial debridement and temporary stabilisation with or without negative pressure dressing, in the presence of a senior plastic surgeon. These patients may return to theatre for further debridement and assessments prior to their definitive operation.

Following these initial procedures, patients were booked into a joint orthoplastic list, with both orthopaedic and plastic surgeons present aiming to achieve a single-stage “fix-and-flap” procedure in the same session as advised by the BOA/BAPRAS guidelines [6]. In this definitive orthoplastic procedure, a minimum of five deep bone and soft tissue samples were taken for extended cultures according to the Oxford protocol [10-12]. Following the procedure, patients were admitted to a plastic ward staffed by specialised nurses experienced in the care and management of flaps and grafts.

After discharge from the hospital, these patients were actively followed up in a joint orthoplastic clinic for at least one year. If any issue related to the operation presented afterwards, the patient’s GP (general practitioner) would refer them back to our service. Throughout their journey from admission/referral to discharge from our service, these patients were managed by a multidisciplinary team involving orthopaedics, plastics, infectious diseases, physiotherapy, occupational therapy, and mental health.

Data collection

The data for this research was collected from inpatient stay notes, referral notes, operative notes, clinic letters, radiological studies and microbiology lab results. Patients’ comorbidities were identified and used to calculate a Charlson comorbidity index (CCI) score for each patient.

Clear definitions of primary outcomes were set prior to data collection. Deep infections were identified as those meeting the definition of the Centers for Disease Control and Prevention (CDC) for deep incisional surgical site infection (SSI) [8]. These infections required either revision surgeries or long-term intravenous antibiotics. Superficial wound infections or pin-site infections were identified as those which showed localised signs of infection but did not meet the CDC definition of deep incisional SSI. They only required a short course of oral antibiotics to resolve. Union was assessed by both clinical and radiological evidence of bone healing. Primary union was defined as a union which was achieved in less than 12 months from the date of the definitive operation. Secondary union was defined as a union which was achieved following further surgical intervention after the definitive operation, whereas a delayed union was that which was achieved after more than 12 months from the date of the definitive operation but without any additional surgeries. Non-union was identified as the absence of signs of fracture healing for at least three consecutive months in a fracture that is at least nine months old (FDA definition of non-union) [13].

Statistical analysis

Data were analyzed using R version 4.1 (R Foundation for Statistical Computing, Vienna, Austria). Exploratory data analysis was done using frequency tables and summary measures like mean, median, range (R), interquartile range (IQR), standard deviation (SD), and normality measures like skewness, kurtosis, Shapiro-walk test, and/or Kolmogorov Smirnov test where necessary. T-test/Mann Whitney U test, and ANOVA/Kruskal-Wallis test were used in hypothesis testing for group differences. Correlation analysis was done between numerical variables. Linear regression was used to investigate factors that influence time to union.

## Results

Demographics

A total of 125 patients with 134 GA type IIIB open fractures were included in this study. They were 86 (69%) males and 39 (31%) females, with a mean age of 42.2 years (R: 9-91). Forty-seven (38%) patients were smokers at the time of initial presentation. Seventy-six (60%) patients scored zero in CCI while only 15 (12%) patients scored 4-6 (highest CCI scores in the study). The mean CCI score was 1.

Site

Seven patients had open fractures in two different sites, and one patient had open fractures in three different sites. The leg was the most common fracture site (64%), followed by the ankle (22%), and the foot (8%). The anatomical sites of tibial fractures varied; 43.7% were on distal tibia, 32.2% on mid-shaft tibia, 12.6% were intra-articular pilon fractures and 11.5% were on proximal tibia (Table 1.)

**Table 1:** Anatomical sites of GA type IIIB open fractures.

Site		Frequency (%)
Leg		86 (64)
	Tibia and fibula	58 (43)
	Tibia	17 (13)
	Pilon	11 (8)
Ankle		30 (22)
Foot		11 (8)
	Mid-foot	7 (5)
	Hind-foot	3 (2)
	Fore-foot	1 (1)
Femur		3 (2)
Forearm		2 (1)
	Radius and ulna	1 (1)
	Ulna	1 (1)
Elbow		1 (1)
Hand		1 (1)
Total		134 (100)

Operative management

The mean time from presentation to initial debridement was 12.4 hours (SD: 7.3). A total of 119 (95.2%) patients had their initial debridement performed within 24 hours. Of these, 62 (49.6%) had it performed within 12 hours. The remaining six patients had it performed after 24 hours of presentation. The mean time from presentation to definitive fix-and-flap surgery was 8.5 days (SD: 7.41, median: 7, IQR: 10.5-4 = 6.5). Twenty-five (20%) patients had their definitive operation performed within 72 hours and 71 (57%) within seven days. The remaining 54 (43%) patients had their definitive operation performed after more than seven days from the time of their presentation. The delay was mainly due to either transfer from other hospitals or the need for further medical optimisation prior to definitive surgery. The most commonly used fixation modalities were open reduction and internal fixation using plates and screws (ORIF) (29.4%), intramedullary nailing (IM nail) (24.6%), circular frame (15.9%) and monolateral external fixation + ORIF (14.3%). Other fixation modalities are shown in Table 2.

**Table 2:** Types of surgical implants used for definitive fixation. ORIF: Open reduction and internal fixation with plates and screws; K-wire: Kirschner wire

Surgical implant	Frequency (%)
ORIF	37 (29.4)
Intramedullary nail	31 (24.6)
Circular frame	20 (15.9)
Monoliteral external fixator + ORIF	18 (14.3)
Circular frame + ORIF	5 (4)
K-wires	3 (2.4)
Monoliteral external fixator	2 (1.6)
Intramedullary nail + ORIF	2 (1.6)
Other fixation modalities	8 (6.3)

Soft tissue coverage in the definitive fix and flap operation was achieved through soft tissue transfer (free flaps) (43.2%), local flaps (40.8%), skin grafts (12.8%), and other modalities (3.2%). Table 3 shows the different modalities used for soft tissue coverage.

**Table 3:** Types of soft tissue coverage used in the definitive operation.

Type of Flap	Frequency (%)
Anterolateral thigh (ALT) flap	51 (41)
Locoregional flaps	49 (39)
Skin graft	16 (13)
Latissimus dorsi flap	3 (2)
Fasciocutaneous flap	1 (1)
Hatchett flap	1 (1)
Others	4 (3)

The median number of operations for these patients from the initial debridement to the latest operation prior to the date of data collection was three operations (IQR: 4-2 = 2). These operations included initial debridement, definitive fixation, adjustment of frames or fixation devices, flap revisions, application/change of negative pressure dressings and removal of metalwork, when applicable.

Follow-up and outcomes

The mean follow-up duration from the time of injury to October 2021 was 43.3 (R: 6-100) months.

Infections and Microbiology

Culture growth of the deep bone/tissue samples that were taken in definitive fix and flap operations was identified in 17 (13.6%) patients. *Staphylococcus epidermidis* was the most common organism detected (four patients). Other identified microorganisms are shown in Table 4.

**Table 4:** Microorganisms grown from deep culture samples taken at time of definitive surgery.

Microorganism	Frequency (%)
Staphylococcus epidermidis	4 (23.5)
Staphylococcus aureus	3 (17.6)
Enterobacter cloacae	2 (11.6)
Klebsiella pneumonia	2 (11.6)
Others microorganisms (20)	1 (5.9) each

Superficial wound infections were detected in 45 (36%) patients and were all successfully treated. The most commonly identified organisms in the wound swabs taken from these superficially infected wounds were *Staphylococcus aureus* (51.1%), *Pseudomonas aeruginosa* (15.6%), and *Enterobacter cloacae* (13.3%). Other identified microorganisms are shown in Table 5.

**Table 5:** Microorganisms grown from wound swab samples of superficially infected wounds.

Microorganism	Frequency (%)
Staphylococcus aureus	23 (51.1)
Pseudomonas aeruginosa	7 (15.6)
Enterobacter cloacae	6 (13.3)
*Haemolytic Streptococcus *(Group B)	3 (6.7)
Klebsiella oxytoca	3 (6.7)
Stenotrophomonas maltophilia	2 (4.4)
Vancomycin-resistant Enterococci	2 (4.4)
Klebsiella pneumoniae	2 (4.4)
Other microorganisms (22)	1 (2.2) each

Three patients (2.4%) developed deep (metalwork) infections that necessitated removal of metalwork and use of local antibiotics. All of them were tibial fractures. Union was achieved in all three afterwards. These three patients had their initial debridement performed in 3, 5-10 hours from the time of presentation. Deep (metalwork) infection was associated with time from presentation to initial debridement (p = 0.049). However, when categorising this time period according to the BOA/BAPRAS recommended thresholds, which are based on the mechanism of injury and level of contamination, there was no statistically significant association between metalwork infection; time to initial debridement being within 12 hours, between 12 and 24 hours, or more than 24 hours (p = 0.340). Also, there was no association between metalwork infection and time to definitive operation; neither as a continuous variable (p = 0.934) nor as categorised to within 72 hours, between three and seven days, or after seven days from initial X-rays (p = 0.589).

Primary outcomes

We excluded 23 patients from calculations of primary outcomes; Five patients were repatriated to their referring hospital following the definitive surgery, 12 patients were lost to follow-up, and six patients died due to other causes not related to the injury, prior to achieving union. Of the remaining 102 patients, primary union was achieved in 64 (62.7%%) patients. Twenty-two (21.6%) patients had delayed union and 3 (2.9%) patients achieved secondary union after further operations following their definitive fix and flap operation. The mean time to union for these three groups of patients (primary union, secondary union, and delayed union) was 10.1 months (SD: 7.23, median: eight months, R: 2-48 months). One patient (1%) was showing progression towards union four months after surgery (one month prior to data collection), and was still under active follow-up.

Non-union was identified in seven (6.9%) patients. Time to union was associated with modality of fixation (p = 0.002) and type of soft tissue coverage (p = 0.028). The group means of time to union for the five most common fixation modalities (representing 84% of all modalities) and for the different types of soft tissue coverage are shown in Tables 6-7. Time to union was also negatively correlated with time from presentation to first debridement (p= 0.002, correlation coefficient: -0.321). Linear regression showed that time to union decreases by 0.27 month for every hour delay in time from presentation to initial debridement (p=0.021).

**Table 6:** Mean time to union (in months) of the most commonly used fixation modalities. ORIF: open reduction and internal fixation with plates and screws.

Modality of fixation	Mean time to union (months)
ORIF	7.7
Intramedullary nail	9.8
Circular frame	17.5
Monolateral external fixator + ORIF	8.4
Circular frame + ORIF	10.5

**Table 7:** Mean time to union (in months) of different types of soft tissue coverage.

Soft tissue coverage (No. of patients)	Mean time to union (months)
Soft tissue transfer (free flaps) (54)	11.7
Local flap (51)	8.1
Skin graft (16)	11.2
Others (4)	9.7

Limb Salvage

Three patients (2.9%) required amputation following their definitive operation. All of them had mid-shaft tibial fractures and required below-knee amputation. Two of them had extensive soft tissue loss and underwent amputation two to four weeks after definitive surgery due to flap failure. The third patient developed unsalvageable flap congestion and necrosis post-operatively and amputation was performed eight weeks after definitive surgery.

Flap Failure

Flap failure that didn’t end up with amputation was reported in two (1.9%) patients. One of them had an anterolateral thigh (ALT) flap for an ankle fracture fixed by ORIF. the flap developed necrosis and required multiple salvage operations including two skin graft operations. The other patient had a Loco-regional flap for distal tibial fracture fixed by an intramedullary nail. The patient developed multiple flap infections and chronic osteomyelitis on the exposed bone. The patient was advised for amputation but refused and preferred to stay on VAC therapy and long-term antibiotics. This patient subsequently died 13 months after his definitive fix and flap operation.

Other than the above-mentioned association between deep (metalwork) infection and time from presentation to initial debridement (p = 0.049), there was no statistically significant association between the primary outcomes (union status, superficial wound infection, metalwork infection, flap failure, limb salvage) and Charlson comorbidity index, smoking status, anatomic site of fracture, time from presentation to first debridement, time from presentation to definitive operation, and modality of fixation or type of soft tissue cover.

## Discussion

The combined orthoplastic approach in the management of GA type IIIB open fractures is vital for both skeletal stability and soft tissue reconstruction. It aims to maximize the functional outcomes of such injuries through meticulous techniques and timely coordinated management [8]. The management of such injuries starts with initial debridement of the devitalized tissues and evaluation of the soft tissue loss to plan ahead for the definitive skeletal fixation and soft tissue coverage. The historical six hours threshold for initial debridement proposed by Kendsfater [14] has been made obsolete by several studies, eg; Sigh et al., which showed no difference in infection rate between patients who had debridement in less than six hours and those who had it in more than six hours [15]. The BOA/BAPRAS standards replaced this threshold by advising on immediate debridement for grossly contaminated wounds or suspected compartment syndrome, debridement within 12 hours for solitary high-energy open fractures and debridement within 24 hours for all other low-energy open fractures [6]. A study of 77 open fractures by Patzakis et al. and another meta-analysis involving over 18,000 patients by Goliath investigators showed increased risk of infection after 12 hours [16,17]. However, Hendrickson et al., in their retrospective review of 116 GA grade IIIB open fractures, showed no difference in infection rates when debridement is done before or after the 12-hour threshold [18].

Hull et al. argued that dividing such a continuous variable into arbitrary thresholds will reduce the power of the statistical tests. They applied association tests without arbitrary thresholds for a series of 365 patients with 459 open fractures and found that every hour of delay to debridement is associated with a small added increase in the likelihood of infection, and this delay has a larger absolute effect on fractures of the tibia, those of higher GA grade and those that are grossly contaminated [19]. Nevertheless, Weber et al. in their prospective review of nearly 8000 patients and Al-Hourani et al. in a level-1 trauma centre both showed no association between time to initial debridement and infection, without using any time thresholds [20,21].

Our study showed that deep (metalwork) infection could be associated with time from presentation to debridement (p = 0.049). The three patients in our series who developed metalwork infection had their debridement in three, five, and 10 hours from the time of presentation. However, when categorizing this time period according to the BOA/BAPRAS recommended thresholds that are based on the mechanism of injury and level of contamination, there was no statistically significant association between metal work infection and time to initial debridement being within 12 hours, between 12 and 24 hours or more than 24 hours (p = 0.340). This can be relayed to the fact that on presentation, grossly contaminated severe fractures with a generally higher baseline risk of infection are usually debrided earlier, thus confusing the relationship between delayed debridement and the development of later infections. However, an injury severity score wasn’t used to categorise these fractures in order to support this assumption.

Another systemic review by Schenker et al. that included a total of 3539 open fractures from six prospective and 10 retrospective cohort studies showed no significant difference in the infection rate between open fractures debrided early or late according to any of the time thresholds used in the included studies, even when compared in subgroups according to the GA classification, level of evidence, depth of infection, and anatomic location [22].

Infective complications of open fractures have been widely researched, as it is a serious complication that affects the functional outcomes of the management of these fractures, increases the direct cost of treatment by over 60%, and nearly doubles the length of stay [23]. To optimize recovery and minimise the risk of infection, a target of achieving definitive internal stabilization that is immediately followed by soft tissue coverage within 72 hours from injury was proposed by Godina who showed a significant difference in infection rate (from 1.5% to 17.5%) between those who had bony and soft tissue reconstruction performed within 72 hours and those who had it performed between 72 hours and three months [24]. This target was later adopted by The BOA/BAPRAS to become the standard of care [6].

Our study showed that the mean time from presentation to the definitive fix-and-flap surgery was 8.52 days with only 25 (20%) patients having their definitive operation performed within 72 hours and 71 (57%) patients within seven days. The rest of the patients (43%) had their definitive operation performed after more than seven days from the time of presentation. The delay was due to either transfer from other hospitals or the need for further medical optimisation prior to definitive surgery. Despite the time from injury to the definitive operation being much longer than the proposed targets set by the BOA/BAPRAS standards, the outcomes of these patients in regards to infection rates and union after primary surgery were very good compared to the historically available literature.

There was no association between time to definitive operation and rate of infection (p= 0.934) or time to union (p= 0.511). The inability to eliminate unchangeable causes of delay (e.g. the need for further medical optimization and poly-traumatic presentation) [25], and the comparable outcomes of patients who had the definitive stabilization and soft tissue cover after 72 hours from injury as in this study and in other studies [8,9,21], shows the outweighing importance of achieving a meticulous and simultaneous ortho-plastic approach over focusing on achieving the 72 hours target if the latter leads to a suboptimal definitive operation.

The mean time to union following primary surgery in this study was 10.1 months. Time to union was negatively correlated with time from presentation to debridement, and linear regression showed that for every hour of delay of debridement, time to union decreases by 0.27 months (p=0.02). Our data would question the significance of the current guidelines with regard to the timing of surgeries in these injuries. It’s obvious that this result cannot be used to advocate for delayed debridement, but it can guide us to understand the higher relevance of Getting It Right First Time (GIRFT) and prioritising the availability of surgical expertise, skilled staff, and equipment over rushing the patient to the theatre for a rapid operation aiming to adhere to these time thresholds. This was also recommended by Nicolaides et al.'s systemic review, which included 20 studies with 10,032 open tibial fractures and similarly didn’t find any statistically significant association between delayed debridement and infection rate or non-union rate [26].

A comparison of the outcomes of GA IIIB open fractures of historical studies from available literature with the current study is given in Table 8.

**Table 8:** Comparing the outcomes of Gustilo–Anderson IIIB open fractures of historical studies from available literature to this study. *including Type IIIB & IIIC only, **including Type IIIA, IIIB & IIIC only, *** calculated for primary union only, w: weeks, m: months.

	Number of patients with GA IIIB fractures	Follow-up (months)	Deep infection rate (%)	Union rate after primary surgery (%)	Mean time to union	Limb salvage rate (%)
Gustilo et al., 1984 [23]	25	26	52	NA	NA	84
Gopal et al., 2000 * [27]	80	NA	9.5	66	25 w	95
Keating et al., 2000 [28]	55	41	17.5	58	43 w	98.2
Naique et al., 2006 [29]	72	41	8.5	NA	28 w	93
Tielinen et al., 2007 [30]	19	10-119	0	53	8 m	100
Rohde et al., 2007 [31]	38	NA	18.4	71	NA	94.7
Singh et al., 2012 [15]	39	>12	20	NA	NA	NA
Hull et al., 2014 [19]	73	>12	20.5	NA	NA	NA
Mathew et al., 2015 ** [8]	73	>12	14.9	NA	NA	91.9
Wordsworth et al., 2016 [32]	65	40	1.6	93.5	33.2 w	94
Doshi et al., 2017 [33]	21	12	2.3 **	NA	24.5 w	NA
Hendrickson et al., 2018 [18]	112	19.7	5.2	NA	NA	NA
Al-Hourani et al., 2019 [21]	45	24	8.9	88.9	NA	100
Aljawadi et al., 2021 [9]	102	25	0.98	86.7	32 w ***	97
Higgin et al., 2021 [34]	116	46	12	84	NA	83
Current study	125	43.3	2.4	84.3	10.1 m	97.1

Limitations

It’s important to mention that some confounding variables might have obscured these results, e.g. baseline contamination, injury severity score (ISS), and use of VAC dressing, and data for these variables should have been collected to optimize our results. Additionally, the number of patients in some subgroups of categorical variables (e.g. time to debridement when categorized according to the BOA/BAPRAS standards) was significantly small, which might affect the statistical power of the association tests. Also, this study has been conducted in an ortho-plastic unit where the simultaneous ortho-plastic fix and flap approach is advocated and this might create a confirmation bias for the outcomes of our technique.

We recommend conducting a bigger scale multi-centre study with a larger number of patients in each subgroup and ensuring to control the confounding variables to get more accurate statistical results. 

## Conclusions

To our knowledge, this is the largest series of GA IIIB open fractures with the longest mean follow-up duration. It shows that low infection rates, high union rates, and high limb salvage rates can be achieved with meticulous technique of simultaneous definitive fixation and soft tissue coverage, coupled with continuous multidisciplinary input.

Our study showed that delaying initial debridement or soft tissue coverage didn’t increase the rate of metalwork infections. The three patients who developed metalwork infections had their initial debridement within 12 hours of injury. It also showed a negative correlation between time to debridement and time to union. We advise on prioritizing surgical technique and availability of expertise over strict adherence to time thresholds if the latter means compromising the former.
